# Predictors of influenza among older adults in the emergency department

**DOI:** 10.1186/s12879-016-1966-4

**Published:** 2016-10-28

**Authors:** Po-Po Lam, Brenda L. Coleman, Karen Green, Jeff Powis, David Richardson, Kevin Katz, Bjug Borgundvaag, Telisha Smith-Gorvie, Jeffrey C. Kwong, Susan J. Bondy, Allison McGeer

**Affiliations:** 1 Dalla Lana School of Public Health, University of Toronto, Toronto, ON Canada; 2 Department of Microbiology, Mount Sinai Hospital, Toronto, ON Canada; 3 Toronto Invasive Bacterial Diseases Network, Toronto, ON Canada; 4 Toronto East General Hospital, Toronto, ON Canada; 5 William Osler Health System, Toronto, ON Canada; 6 North York General Hospital, Toronto, ON Canada; 7 Schwartz/Reisman Emergency Medicine Institute, Mount Sinai Hospital, Toronto, ON Canada; 8 Department of Emergency Medicine, University Health Network, Toronto, ON Canada; 9 Institute for Clinical Evaluative Sciences, Toronto, ON Canada

**Keywords:** Influenza, Older adults, Elderly, Clinical symptoms

## Abstract

**Background:**

Diagnosis of influenza in older adults may be complicated by atypical presentations or when patients present with complications of an underlying illness. We aimed to identify clinical characteristics and epidemiological factors associated with influenza among community-dwelling adults aged ≥60 years presenting to emergency departments.

**Methods:**

We identified patients with influenza-compatible chief complaints presenting to emergency departments of six acute care hospitals in Ontario, Canada during the 2011/12 and 2012/13 influenza seasons. Clinical characteristics, medical history and demographics were collected by patient interview, chart review and by contacting vaccine providers. Nasopharyngeal swabs were tested for influenza using polymerase chain reaction. We modeled predictors of influenza using multivariable logistic regression models that compared individuals with and without influenza.

**Results:**

Of 1318 participants, 151 (11 %) had influenza (98 A/H3N2, 12 A/H1N1, 4 A [not sub-typed], 37 B). In the multivariable model, clinical symptoms associated with influenza were cough (OR 6.4, 95 % CI 3.2, 13.0), feverishness and/or triage temperature ≥37.2 °C (OR 3.0, 95 % CI 2.0, 4.7), 2–5 days from symptom onset to the emergency department visit (OR 2.2, 95 % CI 1.5, 3.2), and wheezing (OR 2.1, 95 % CI 1.3, 3.3). The effect of cough on influenza increased with older age. Epidemiological factors associated with increased odds for influenza included weeks when ≥10 % influenza tests from provincial laboratories were positive (OR 5.1, 95 % CI 1.2, 21.7) and exposure to a person with influenza-like illness (OR 1.9, 95 % CI 1.3, 2.8). Among participants with influenza, only 47 (31 %) met the U.S. Centers for Disease Control and Prevention criteria for influenza-like illness (temperature ≥37.8 °C and cough and/or sore throat).

**Conclusions:**

As in younger adults, cough and feverishness are the two symptoms most predictive of influenza in the elderly. Current influenza-like illness definitions did not adequately capture influenza in older adults.

## Background

Accurate and timely diagnosis of influenza is especially important for older adults who are at higher risk for severe complications and for whom influenza vaccines are less effective [1–3]. Laboratory testing with polymerase chain reaction (PCR) is not always available, and most other tests are of relatively low sensitivity and/or may not yield results in a timely manner [4]. Diagnosis based on signs and symptoms is challenging because influenza symptoms are difficult to distinguish from those of other viral respiratory tract infections (e.g., respiratory syncytial virus, rhinovirus) [2, 5], and because influenza may present as a complication of an underlying illness (e.g., exacerbation of chronic obstructive pulmonary disease) rather than as a primary illness [2].

Current evidence on the predictors of influenza in the elderly is limited. Previous studies have identified feverishness, cough, myalgia, pain on respiration, headache, sore throat and rigor/chills as clinical symptoms associated with influenza [6–12]. As in younger adults, cough and fever have the highest positive predictive value (PPV) [13]. However, past efforts to identify useful symptom clusters have not been able to determine a combination that discriminate adequately between influenza and other illnesses [6, 8, 11, 12, 14]. The aim of this study was to identify clinical characteristics and/or epidemiological factors associated with influenza among community-dwelling adults older adults presenting to emergency departments (EDs).

## Methods

### Study design and setting

We prospectively recruited community-dwelling older adults age ≥60 years presenting to the EDs of six acute care hospitals in urban and suburban areas of south-central Ontario, Canada. These EDs had a median of 175 visits per day (range 160–350). Patients or their substitute decision-makers were approached for consent, nasopharyngeal swabs were collected, and, whenever possible, interviews were conducted while they were in the ED.

### Participant enrollment

**Fig. 1:**
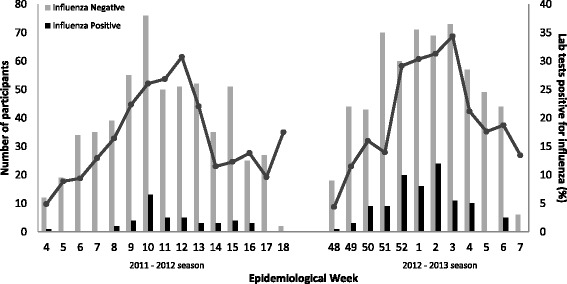
Participants with and without influenza and the percentage of specimens testing positive for influenza submitted to Ontario laboratories by epidemiological week, 2011–2012 and 2012–2013 seasons

Participant enrollment occurred during weeks when the proportion of specimens submitted to Ontario laboratories testing positive for influenza was greater than 5 % for the 2011/12 and 2012/13 seasons (Fig. 1). Study personnel screened and enrolled patients from each site during five, 8-h shifts per week. The shifts were scheduled to sample patients in each ED on all seven days of the week for 24 h of the day. Study eligible patients who had most recently arrived were approached first by the research assistant to avoid bias introduced by recruiting patients with longer ED stays. Study approval was obtained from all participating hospital research ethics boards (REBs).

Eligible patients had chief complaints of cardiac, respiratory or cerebrovascular events, systemic symptoms (fever, weakness) or altered level of consciousness. Patients with chief complaints related to trauma, mental disorders, post-operation complications and/or urogenital problems were not eligible. For other chief complaints, patients were included if their triage record indicated any of the following symptoms or conditions: respiratory symptoms, triage temperature ≥37.5 °C, feverishness, malaise, or general weakness. Residents of long-term care homes or other group settings (e.g., detention centers) and patients who had been enrolled in the study within the previous 14 days were not eligible.

### Data collection

Recruiters interviewed participants to determine symptoms, lifestyle, healthcare use, history of pneumococcal vaccination, and influenza vaccination in the previous five years, medical care/consultation during current illness episode, and Clinical Frailty Scale items [15]. Demographic, medical history, and symptom information were also collected by chart review. The participant’s vaccine provider and/or family physician was contacted to obtain influenza vaccination history (for the present and previous season), pneumococcal vaccination history and information on prior visits related to the current episode of illness. Vaccine providers were re-contacted to confirm vaccination information if provider and patient-reported status were discrepant. If the discrepancy could not be resolved, the ‘yes’ response from either source was accepted. Similarly, for discrepancies between self-reported and chart data for symptoms or medical history, a ‘yes’ from either source was taken as the final response.

The outcome of interest was laboratory-confirmed influenza infection. A nasopharyngeal swab collected on date of enrolment was tested for influenza by reverse transcription PCR (RT-PCR) at a single study laboratory within 24 h of collection using bioMérieux easyMAG with RealStar® influenza RT-PCR (Altona Diagnostics) in 2011/12 and Simplexa™ Flu A/B & RSV Direct RT-PCR (Focus Diagnostics) in 2012/13.

*A priori* hypothesized predictors of influenza infection were symptoms (cough, fever, acute confusion, sore throat, chills, headache, myalgia, rhinorrhea, wheezing), acute onset of symptoms, influenza vaccination status, children aged ≤16 years in the household, regular contact with children outside the household, exposure to person(s) with influenza-like illness (ILI), recent use of public transit, and the level of influenza activity in the community. We also hypothesized that the association between symptoms and influenza infection would vary across age, presence of underlying illnesses, use of medications for the current episode of illness, deprivation score, and level of frailty.

### Predictor definitions

We chose definitions of predictors to maximize sensitivity and best discriminate between those with and without influenza, with preference given to variables being readily available in ED settings. We assessed self-reported and chart-recorded severity (mild, moderate or severe) of clinical symptoms as well.

The definition of cough was a composite of self-reported and/or chart-recorded data. Participants were recorded as having cough if it was reported during the interview as a symptom prior to the ED visit or if cough was recorded in the medical chart. Fever was defined as self-reported feverishness and/or having a triage-recorded temperature ≥37.2 °C [13]. All participants were asked if they felt feverishness during their current episode of illness. Acute onset of symptoms was defined as being sudden (i.e., woke up feeling miserable or were unable to do regular activities within a few hours) versus gradual development (i.e., symptoms worsened over several days). Time from symptom onset to ED visit was calculated as the number of days between participant-reported day of onset and presentation to the ED.

A participant was defined as having received the influenza vaccine if it was received in the current season and ≥14 days before symptom onset. Participants' chronic conditions were abstracted from medical records and medication history was collected by interview and medical chart review.

Participants were asked if they lived with children (≤16 years), and whether they had regular contact (≥4 h per day and for ≥4 days per week) with other children outside the household. Recent use of public transportation included riding a bus, subway, train, or streetcar within four days of symptom onset. Exposure to a person with an ILI was defined as being within arm’s length for ≥2 min to someone the participant recognized as having symptoms of an acute respiratory illness (i.e., had cough, stuffy or runny nose, hoarseness or a sore throat) within 7 days of symptom onset.

Influenza activity in the community was defined as the proportion of specimens positive for influenza submitted to laboratories in south-central Ontario during the past week. Neighborhood-based material and social deprivation indices were determined by matching participants’ postal codes with the Canadian Deprivation Index database [16]. The index is divided into 5 quintiles with ‘1’ being the most privileged and ‘5’ representing the most deprived.

Ethnicity was self-reported by participants. Frailty was defined as having a score of ≥5 on the Clinical Frailty Scale (maximum 9 points), as scored by a trained research assistant [15], where the participant required at least some assistance with daily activities [17].

### Statistical analyses

We compared the characteristics of individuals having laboratory-confirmed influenza with those who tested negative. Chi-square and Fisher’s exact tests were applied to assess differences among group proportions. Medians were compared using Wilcoxon rank-sum tests. Reported *p*-values are two-sided with a value <0.05 considered to be statistically significant. No correction was made for multiple testing. Odds ratios (OR) and 95 % confidence intervals (CI) were estimated using logistic regression. Hypothesized predictors significant at p <0.2 in the bivariate analysis were included in the initial logistic regression model [18]. The full model was reduced by sequentially removing predictors that did not significantly improve the fit of the model as determined by the Likelihood Ratio Test [18]. Hosmer-Lemeshow Goodness-of-Fit Test and residuals plots were used to assess model fit. Variance inflation factors (VIF > 10) were used to assess for collinearity between predictors. No imputation was done for missing data. In the multivariable analysis, participants with missing data on included predictors were excluded. The final model was adjusted for hospital site and had at least ten events per predictor in the model.

Statistical interaction terms, determined *a priori*, were entered as cross-product terms in a logistic regression model. If significant in the crude analysis, the interaction term was added to the final model and assessed for significance [19]. To interpret effect modification, the odds ratio was calculated for one predictor on the outcome over the levels of another predictor. Vaccine effectiveness was calculated as (1 - OR) X 100.

To explore potential differences between influenza types (A & B), we compared each type to influenza-negative participants. We used SAS version 9.3 (SAS Institute, Cary, NC, USA) for all analyses.

## Results

### Participants

**Fig. 2:**
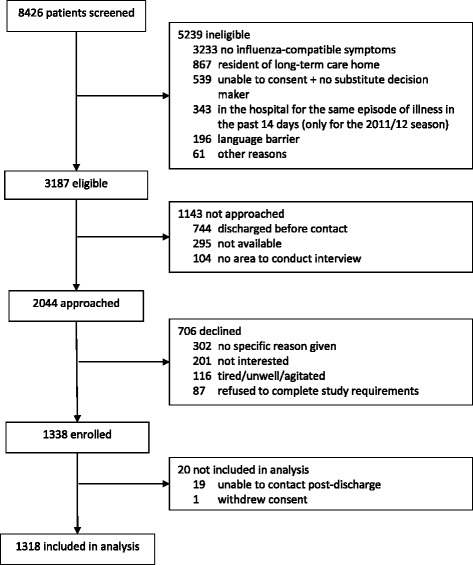
Flow diagram of participant enrollment

Among eligible patients, 64 % were approached by a research assistant and, of those approached, 65 % consented to participate in the study (Fig. 2). In total, 1318 people were enrolled: 606 (46 %) during the 2011/12 and 712 (54 %) during the 2012/13 influenza seasons. Median participant age was 76.4 years (interquartile range: 68.5–84.2 years), and 656 (50 %) were female. The majority (*n* = 868, 66 %) had been immunized against influenza in the current season, and 61 % reported one or more underlying chronic diseases. Overall, 151 participants (11 %) tested positive for influenza (98 A/H3N2, 12 A/H1N1, 4 A [not sub-typed], 37 B). A greater proportion of individuals had influenza during the 2012/13 season (108/712, 15 %) compared to the 2011/12 season (43/606, 7 %; *p* < 0.001). The proportion of participants with influenza varied across hospital sites ranging from 6 % to 23 % (median 18 %).

Participants with influenza and those without influenza did not differ by age, sex, vaccination status, or underlying chronic disease status (Table 1). However, those with influenza were significantly more likely to live in neighborhoods with high material deprivation scores, less likely to be Caucasian, and more likely to have been prescribed antibiotics and used symptom relief medications prior to ED arrival. Only two participants reported the use of an antiviral drug prior to their ED visit.

**Table 1:** Symptoms and characteristics of participants aged ≥60 years with and without influenza presenting to emergency departments, Ontario, Canada, 2011/12 and 2012/13

Participant characteristics/symptoms^a^	No Influenza	Influenza	*p*-value
	*n* = 1167 (%)	*n* = 151 (%)	
Age, median (IQR)	76.1 yrs (IQR 68.4–84.1)	77.4 yrs (IQR 68.9–86.1)	.35
Female sex	578 (50)	78 (52)	.62
Used public transportation ≤4 days before onset	160 (14)	23 (15)	.62
Children (≤16 years) in household	98 (8)	17 (11)	.26
Works with children	14 (1)	5 (3)	.06
Exposed to person with ILI ≤7 days before onset	275 (24)	64 (42)	<.001
Material deprivation index^†^, Median	3 (IQR 1–4)	3 (IQR 2–5)	.001
White/Caucasian	895 (78)	105 (70)	.03
Influenza vaccine, current season	768 (66)	100 (68)	.75
Pulmonary /cardiac disease or immunosuppressed	709 (61)	93 (62)	.84
Frail (≥5 on Clinical Frailty Score)	309 (27)	36 (24)	.35
Enrolment season:			
2011/12	563 (93)	43 (7)	<.001
2012/13	604 (85)	108 (15)	
Enrolled week when ≥10 % of influenza tests positive	1057 (91)	149 (99)	<.001
Healthcare use prior to ED arrival			
Prior consultation with health practitioner	465 (40)	66 (44)	.34
Antibiotics prescribed	187 (16)	40 (26)	.002
Antipyretics taken	512 (44)	68 (45)	.79
Other general symptom relief medications taken	85 (7)	24 (16)	.001
Presenting symptoms			
Cough (self-report and/or chart)	649 (56)	142 (94)	<.001
Wheezing (chart)	90 (8)	35 (23)	<.001
Sore throat	244 (21)	60 (40)	<.001
Stuffy/runny nose	353 (30)	88 (58)	<.001
Sneezing	274 (24)	50 (33)	.01
Shortness of breath	693 (59)	98 (65)	.19
Any one or more of: sore throat, runny/stuffy nose or sneezing	522 (45)	111 (74)	<.001
Feverishness and/or triage temp ≥37.2 °C	463 (40)	115 (76)	<.001
Headache	419 (36)	71 (47)	.01
Myalgia	357 (31)	71 (47)	<.001
Chills (self-report and/or chart)	172 (15)	57 (38)	<.001
Weakness	873 (75)	125 (83)	.03
Fatigue	906 (78)	138 (91)	<.001
Loss of appetite	603 (52)	102 (68)	<.001
Median number of systemic symptoms (headache, myalgia, chills, weakness, fatigue, or loss of appetite)	2 (IQR 2–3)	3 (IQR 2–4)	<.001
Confused (self-report and/or chart)	257 (22)	46 (30)	.02
Time from symptom onset to ED triage:			
0–1 day	451 (39)	25 (17)	<.001
2–5 days	320 (27)	81 (54)	
≥ 6 days	393 (34)	43 (29)	
Acute onset of symptoms^‡^	586 (50)	70 (47)	.33

*IQR* Interquartile range, *ED* Emergency department, *ILI* influenza-like illness

^a^Symptoms and characteristics were based on self-reported data unless indicated otherwise; The proportion of participants with missing data for any given predictor did not exceed 3 %

^†^Material deprivation index ranks from 1 (most privileged neighborhood) to 5 quintiles (most deprived neighborhood)

^‡^Acute onset was self-defined as symptom development within a few hours rather than gradual worsening of symptoms ≥ 6 h

Among those with influenza, 31 % presented with symptoms that matched the U.S. Centers for Disease Control and Prevention definition for ILI (temperature ≥37.8 °C and cough and/or sore throat) [20], with a specificity of 92 %, sensitivity of 31 %, PPV of 34 % and negative predictive value (NPV) of 91 %. The Public Health Agency of Canada definition for ILI (acute onset of symptoms, fever and cough with sore throat, arthralgia, myalgia or prostration) [21] captured 32 % of the influenza positive participants with a specificity of 91 %, sensitivity of 32 %, PPV of 31 % and NPV of 91 %.

### Predictors of influenza

Cough as indicated by medical chart review had lower sensitivity (128/151, 85 %) compared with self-report (139/151, 92 %). A composite variable that included self-reported and/or chart-recorded cough achieved the highest sensitivity (142/151, 94 %) with 44 % specificity, 18 % PPV and 98 % NPV. Cough was the most common (94 %) respiratory symptom among those with influenza (Table 1). In the bivariate analysis, all three definitions of coughing were associated with influenza: cough as indicated by chart review (OR 8.9, 95 % CI 5.6, 14.1), self-reported cough (OR 11.1, 95 % CI 6.1, 20.2) and the composite variable (OR 12.6, 95 % CI 6.4, 24.9).

Participants with influenza had a higher median triage temperature than those without influenza (37.3 °C versus 36.7 °C, *p* < 0.001). In this sample, 32 % of those with influenza had a triage temperature ≥37.8 °C (specificity 75 %); a lower temperature cut-off of 37.2 °C captured 55 % of those with influenza (specificity 79 %). A composite variable of self-reported feverishness and/or measured temperature (≥37.2 °C) had the highest sensitivity (76 %), with 60 % specificity, 20 % PPV and 95 % NPV.

Participants with influenza were more likely to report one or more of: wheezing, sore throat, stuffy/runny nose, shortness of breath, or sneezing (Table 1). They were also significantly more likely to report more systemic symptoms (headache, myalgia, chills, weakness, fatigue, or loss of appetite) (*p* < 0.001).

Individuals with influenza were more likely to present to the ED during weeks with a higher proportion of lab specimens yielding influenza as compared to those without influenza (median 29 % versus 21 %, *p* <0.001, Fig. 1). To guide clinical decision-making, a threshold of 10 % was used to define seasonal influenza activity [22].

In the multivariable model (Table 2), the predictors with the highest ORs for influenza were cough and feverishness. Hospital site did not improve the fit of the regression model (likelihood ratio test *p* = 0.12) but it was included in the final model to control for possible confounding on the association between predictors and outcome [18].

**Table 2:** Results of final logistic regression model for influenza, Ontario, Canada, 2011/12 and 2012/13

Predictors^a^	Unadjusted OR (95 % CI)		Adjusted OR^†^(95 % CI)	
	*n* = 1313	*p*-value	*n* = 1313	*p*-value
Presenting symptoms^‡^				
Cough (self-report and/or chart)	12.4 (6.3, 24.6)	<.001	6.4 (3.2, 13.0)	<.001
Wheeze (chart)	3.7 (2.4, 5.7)	<.001	2.1 (1.3, 3.3)	.003
Any one or more of: sore throat, runny/stuffy nose, or sneezing	3.5 (2.4, 5.1)	<.001	–	
Feverishness and/or triage temperature ≥37.2 °C	4.8 (3.2, 7.1)	<.001	3.0 (2.0, 4.7)	<.001
Number of systemic symptom(s)^₴^	1.5 (1.3, 1.7)	<.001	–	
Confused (self-report and/or chart)	1.6 (1.1, 2.3)	.02	–	
Time to ED from symptom onset (2–5 days)^¶^	3.1 (2.2, 4.5)	<.001	2.2 (1.5, 3.2)	<.001
Medical history				
Influenza vaccine, current season	1.1 (0.8, 1.6)	.59	–	
Antibiotics prescribed/taken prior to visit	1.9 (1.3, 2.8)	.001	–	
General symptom relief medications prior to visit	2.4 (1.5, 4.0)	<.001	–	
Frail (≥5 on Clinical Frailty Score)	0.78 (0.52, 1.2)	.24	–	
Other factors				
Age, per 10 year increase^ǁ^	1. 1 (0.9, 1.3)	.51	1.2 (1.0, 1.5)	.03
Children (<16 years) in household	1.4 (0.8, 2.4)	.21	–	
Recent transportation use	1.1 (0.7, 1.8)	.58	–	
Exposed to person with ILI ≤7 days before onset	2.5 (1.7, 3.5)	<.001	1.9 (1.3, 2.8)	.002
Material deprivation index^b^	1.2 (1.1, 1.4)	.002	–	
Caucasian/White	0.67 (0.46, 0.98)	.04	–	
Enrolled week when ≥10 % of influenza tests positive	7.7 (1.9, 31.3)	.005	5.1 (1.2, 21.7)	.03

*OR* Odds ratio, *CI* Confidence interval, *ED* emergency department, *ILI* influenza-like illness

^a^Participants with missing data on included predictors were excluded from the analysis. The proportion of participants with missing data for any given predictor did not exceed 3 %

^b^Material deprivation index ranks from 1 (most privileged neighborhood) to 5 quintiles (most deprived neighborhood). The index is modeled as a continuous variable with one unit increase equivalent to an increase in quintile

^†^Adjusted model also includes hospital site

^‡^Symptoms were based on self-reported data unless indicated otherwise

^₴^Systemic symptoms were headache, myalgia, chills, weakness, fatigue, and loss of appetite. Predictor modeled as a continuous variable with one unit increase per addition of one symptom

^¶^Reference group: 0–1 days and >5 days for time to ED from symptom onset

^ǁ^Age was modeled as a continuous variable per 10 years increase

The OR for the association between cough and influenza infection was modified by age, with the effect of cough being greater in older age groups (Table 3). Among those who were not frail, confusion (as identified by either patient interview or chart review) was associated with having influenza (adjusted OR 2.0, 95 % CI 1.2, 3.3; *p*-value for interaction term = 0.004); no association was observed in frail individuals (adjusted OR 0.46, 95 % CI 0.18, 1.1). The effect of influenza vaccination differed across the two study seasons (Breslow-Day test for homogeneity *p* = 0.01). The estimated vaccine effectiveness (adjusted for predictors and interaction terms in the final model) was 47 % (95 % CI -8 %, 74 %) in 2011/12 and -51 % (95 % CI -154 %, 11 %) in 2012/13, a year with high H3N2 activity.

**Table 3:** Effect of cough on being influenza positive by age, Ontario, Canada, 2011/12 and 2012/13

Age (years)	Odds ratio for influenza when cough is present (95 % CI)^a^
60	1.3 (0.4, 4.6)
65	2.2 (0.9, 5.7)
70	3.8 (1.8, 8.2)
75	6.7 (3.0, 14.9)
80	11.6 (4.1, 33.2)
85	20.1 (4.9, 81.9)
90	34.8 (5.8, 210.6)

*CI* Confidence interval

^a^Odds ratio was adjusted for all other predictors in the final model: hospital site, wheezing, feverishness, confusion, frailty, time to triage from symptom onset, exposure to person with influenza-like illness, influenza activity in the community, product term of confusion and frailty, and product term of cough and age. Cough was based on chart-review and self-report

Almost all influenza B cases (89 %) were recruited in the first season and the majority of influenza A (91 %) cases were from season two. In an exploratory bivariate analysis, cough, wheezing, feverishness, any upper respiratory symptoms and chills were more prevalent among participants with influenza A as compared to those with influenza B. In our sample, predictors of influenza type was confounded by season of enrolment, as participants in season one were more likely to be hospitalized compared to those recruited in season two (70 % versus 50 %, *p* = 0.02).

## Discussion

In this prospective cohort study of community-dwelling older adults aged ≥60 years who attended an ED, we found that symptoms of cough, feverishness, and wheezing were independently associated with having influenza. We also identified independent epidemiological predictors: influenza positivity in Ontario laboratory specimens of ≥10 %, recent exposure to person(s) with ILI, presenting to the ED 2-5 days after symptom onset and advanced age.

Most participants with influenza had cough, which has also been commonly reported in other studies of older adults seeking medical attention [9, 10, 12]. Cough was one of the two strongest predictors of influenza in older adults. Only one other study has identified cough to be a predictor of influenza infection in older adults and with a similar magnitude of effect [6]. In studies that reported a null association [8–11], only participants with ILI were selected, and, as a result, these studies had limited ability to detect the discriminating effect of cough. Cough has also been reported as a clinical predictor of influenza among younger adults and within the same effect size range [13, 23, 24].

In our sample, cough was only associated with influenza among those at advanced ages. The effect of age on the association between cough and influenza is likely related to the combination of the decrease in cough strength with advancing age, and the greater cough stimulus associated with influenza [25].

As seen in previous studies [6–8, 11, 12], fever (self-reported feverishness and/or measured temperature) has been associated with having influenza among older adults. Measured fever is a known predictor of influenza in younger adults [13, 24] but with a higher temperature threshold (≥37.8 °C). It is expected that older adults with acute infections present with a lower febrile response as compared with younger adults. Similarly, a previous study on hospitalized patients aged ≥65 years found that the temperature threshold ≥37.2 °C captured 78 % of influenza-positive individuals [26] .

Participants with influenza in our sample were more likely to present to the ED 2–5 days after symptom onset. Similarly, in other studies of community-dwelling adults, patients with influenza most commonly sought healthcare 3–7 days after symptom onset [8, 27]. A recent meta-analysis showed that early treatment (within two days of symptom onset) with neuraminidase inhibitors is associated with decreased mortality risk compared to later treatment [28]. In our sample, 54 % of those with influenza presented too late to receive such early treatment. Improved outcomes for the majority of our participants will require better prevention, a significant change in patient behaviour, and/or treatment options that are effective when started ≥48 h after symptoms onset.

Confusion is a known predictor of acute infection in older adults [29]. In our sample, confusion was only associated with influenza in those who were non-frail, and not among already frail older adults.

Historically, influenza A has been considered to cause more severe disease as compared to influenza B [30]. More recent evidence has shown that influenza B is also capable of causing severe outcomes [30, 31] and similar clinical symptoms in adults [32]. We found no distinguishing symptoms between influenza types in older adults. However, it should be noted that the power to identify differences is limited by our relatively small sample size.

Exposure to persons with ILI is a known risk factor for infection as human-to-human transmission occurs readily with influenza viruses [33]. Regular contact with children was not a predictor of influenza in our study. However, <10 % of our participants had household exposure to any children, and <3 % had household exposure to children ≤5 years of age. Exposure to children may be a more important factor in populations with different social structures than our study population.

As seen in previous studies, ILI definitions only identified a small proportion of older adults with disease [34]. Syndromic diagnosis of influenza among older adults remains a challenge and delays timely identification of disease in a healthcare setting where the virus can be transmitted to staff and other patients.

Strengths of our study include the use of PCR testing for influenza (for high sensitivity), prospective data collection on a broad range of epidemiological and clinical risk factors, and broad inclusion criteria designed to minimize missed influenza patients associated with selection bias.

There are also a number of limitations. Patients with severe disease or more frail individuals may have been more likely to refuse (e.g., decline because of fatigue) or less likely to be approached for inclusion in the study (e.g., patients requiring resuscitation). However, we interviewed family members of those unable to answer whenever possible, and, among those approached, only 6 % declined because they were too tired or unwell. We were not able to consider the impact of vaccination in prior years on presentation, as the data were often missing, and could not be validated.

We are unable to explain differences in rates of influenza between hospital sites; however, none of our study findings changed when the analysis was limited to specific hospitals, or when hospital site was removed from the multivariable model. To reduce interview time for ill patients, we did not use detailed tools for the assessment of confusion, which may have altered our findings. Because there is no appropriate method to account for multiple testing in an exploratory study [35], results are presented without multiplicity adjustments and maybe therefore be prone to Type I error. Our study is limited to two influenza seasons in one geographic area and to individuals presenting to EDs.

In our sample, exposures differed among those who presented to the ED as their first healthcare contact compared to those who consulted with other healthcare providers prior to their ED visit. Patients presenting to EDs may be different in the severity of illness or other characteristics to those presenting at other types of settings (e.g., primary care, walk-in clinics). The exposures to influenza and care-seeking behavior of older adults are likely to vary between populations.

## Conclusion

In summary, in our population of ED patients, predictors for influenza in older adults are similar to predictors previously identified in younger adults, with the exception that “fever” is better defined using a lower triage temperature threshold. Current clinical definitions of influenza do not adequately capture influenza in older adults. Future research should focus on determining a useful clinical decision rule for influenza in older adults.

## Abbreviations

CI: Confidence interval
ED: Emergency department
ILI: Influenza-like illness
IQR: Interquartile range
NPV: Negative predictive value
OR: Odds ratio
PCR: Polymerase chain reaction
PPV: Positive predictive value
REB: Research ethics board
RT-PCR: Reverse transcription polymerase chain reaction
VIF: Variance inflation factor
